# Leveraging Nuclear Receptors as Targets for Pathological Ocular Vascular Diseases

**DOI:** 10.3390/ijms21082889

**Published:** 2020-04-21

**Authors:** Pei-Li Yao, Jeremy Peavey, Goldis Malek

**Affiliations:** 1 Duke Eye Center, Department of Ophthalmology, Duke University School of Medicine, Durham, NC 27503, USA; Peili.yao@duke.edu (P.-L.Y.); jeremy.peavey@duke.edu (J.P.); 2 Department of Pathology, Duke University School of Medicine, Durham, NC 27503, USA

**Keywords:** nuclear receptors, angiogenesis, wet age-related macular degeneration, proliferative diabetic retinopathy, inflammation

## Abstract

Vasculogenesis and angiogenesis are physiological mechanisms occurring throughout the body. Any disruption to the precise balance of blood vessel growth necessary to support healthy tissue, and the inhibition of abnormal vessel sprouting has the potential to negatively impact stages of development and/or healing. Therefore, the identification of key regulators of these vascular processes is critical to identifying therapeutic means by which to target vascular-associated compromises and complications. Nuclear receptors are a family of transcription factors that have been shown to be involved in modulating different aspects of vascular biology in many tissues systems. Most recently, the role of nuclear receptors in ocular biology and vasculopathies has garnered interest. Herein, we review studies that have used in vitro assays and in vivo models to investigate nuclear receptor-driven pathways in two ocular vascular diseases associated with blindness, wet or exudative age-related macular degeneration, and proliferative diabetic retinopathy. The potential therapeutic targeting of nuclear receptors for ocular diseases is also discussed.

## 1. Introduction

The formation of vascular networks is an essential mechanism that occurs throughout the body and one that has been studied extensively not only in the course of development but also during the initiation and progression of degenerative diseases [1]. As such, it plays an important role in promoting and/or limiting the impact of the inflammatory response [2]. The field of vascular biology often refers to ‘angiogenesis’ as the sprouting of endothelial cells from an existing vascular tree resulting in new vessel formation [3], while ‘vasculogenesis’ denotes new vessel formation during embryonic development. ‘Vasculogenesis’ also occurs in adults and involves the revascularization or neovascularization of damaged tissue. In the ocular field, neovascularization denotes the latter process and occurs in vision debilitating retinal diseases including but not limited to the wet form of age-related macular degeneration and proliferative diabetic retinopathy. Regardless of the blood vessel formation mechanism, key events that occur in vascular development include activation, migration, proliferation, and the maturation of precursor cells [4]. Critical regulators of vascular processes are actively under investigation and are considered important therapeutic targets for vascular diseases. One set of regulators of vascular biology and physiology are a family of transcription factors called nuclear receptors, which have been shown to play an important role during development, aging, and diseases characterized by vascular structure and function abnormalities [5]. The goal of this review is to discuss the potential role of nuclear receptors in ocular vascular diseases of the posterior pole, specifically wet age-related macular degeneration and diabetic retinopathy, which are two leading causes of vision impairment in the elderly and working class populations, respectively.

## 2. Role of Nuclear Receptors in Angiogenesis 

Nuclear receptors (NR) are the largest family of transcription factors in the human genome. Since their discovery in 1988, they have been studied extensively not only in endocrine organs but also in almost all other tissue types within the body [6]. The NR superfamily is comprised of 48 members, and it is functionally diverse. A myriad of mechanisms of action have been linked to the different NRs during normal development, reproduction, and aging as well as the pathology of a number of human neurodegenerative and systemic diseases. Given the importance of blood vessel formation in health and disease, the functions of NRs in angiogenesis and vasculogenesis have also received much attention, in particular in the field of cancer biology [7,8,9,10]. The role of vascular biology in ocular diseases has also stimulated an interest in investigating NRs during ocular development and ocular vascular diseases. Herein, we will focus on a number of seminal studies that have launched an ever-growing interest in understanding the contributions of NRs to ocular angiogenesis and vasculogenesis and in particular the goal of harnessing the power of targeting NRs for potential therapy.

## 3. Nuclear Receptor Signaling in the Pathogenesis of Age-Related Macular Degeneration 

### 3.1. Overview of Age-Related Macular Degeneration

**Figure 1:**
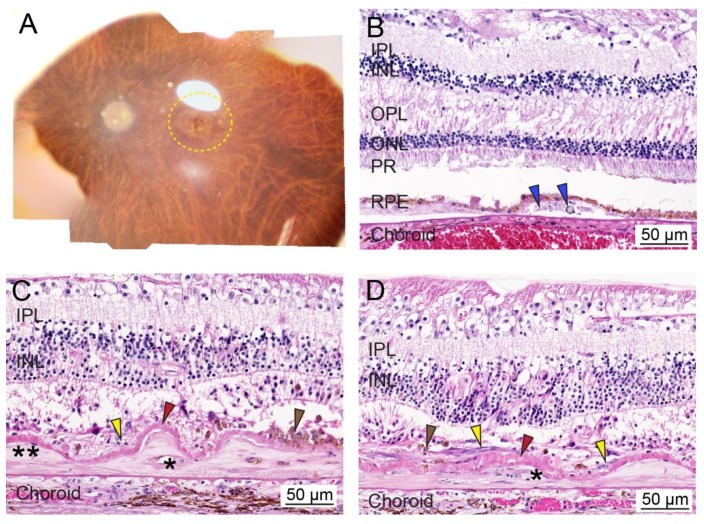
Ex vivo whole tissue and cross-sectional histopathology of human age-related macular degeneration (AMD). (**A**) Representative post-mortem fundus image of an eye from an AMD patient with early dry AMD (OD; 90-year-old male; death to recovery: 7 h 19 min; cause of death: congestive heart failure). The area with AMD lesions is delineated with a yellow dotted line. (**B**) Representative histopathology of paraformaldehyde-fixed paraffin-embedded cross-sections of the retina stained with hematoxylin and eosin from a patient with dry AMD phenotypes (OD; 100-year-old female; death to recovery: unknown; cause of death: unknown). Blue arrowhead: lipid- and protein-rich deposits or drusen within the sub-RPE region. (**C**,**D**) Representative histopathology of paraformaldehyde-fixed paraffin-embedded cross-sections of the retina stained with hematoxylin and eosin from a patient with glaucoma and wet AMD (OD; 103-year-old female; death to recovery: unknown; cause of death: unknown). Severe photoreceptor degeneration, along with two-component fibrocellular disciform scars and a thickened intra-Bruch’s membrane component (two asterisks) and the thin subretinal component (yellow arrowheads). Thin subretinal pigment epithelial fibrovascular membranes (asterisks) are present in disciform scars (two asterisks). A layer of basal laminar deposit (red arrowheads) is located between the disciform scar (yellow arrowheads) and Bruch’s membrane. Brown arrowhead; cluster of pigmented cells. IPL, inner plexiform layer; INL, inner nuclear layer; OPL, outer plexiform layer; ONL, outer nuclear layer; PR, photoreceptor; RPE, retinal pigment epithelium. Bar = 50 µm.

Age-related macular degeneration (AMD), a leading cause of central vision loss in the elderly, is characterized by the accumulation of lipid- and protein-rich deposits between the retinal pigment epithelial (RPE) cells and Bruch’s membrane [11,12]. The advanced clinical sub-types of AMD are differentiated as dry (late dry AMD or geographic atrophy-GA) or exudative (wet AMD) and distinguished by the absence and presence of blood vessels invading from the choroid into the subretinal space, respectively. Clinically, approximately 85% of AMD patients are diagnosed with GA. These patients experience vision loss in the central macula and morphologically present with widespread RPE cell death along with significance photoreceptor loss. Meanwhile, the approximately 10% of patients diagnosed with wet AMD experience choroidal neovascularization (CNV), where immature blood vessels in the choroid expand below the RPE cell layer and often toward the outer retina, resulting in plasma exudation and hemorrhage. Late wet AMD can further evolve into fibrotic scaring, RPE detachment, and acute blindness (Figure 1). 

### 3.2. Molecular Mechanisms and Etiology of AMD 

**Figure 2:**
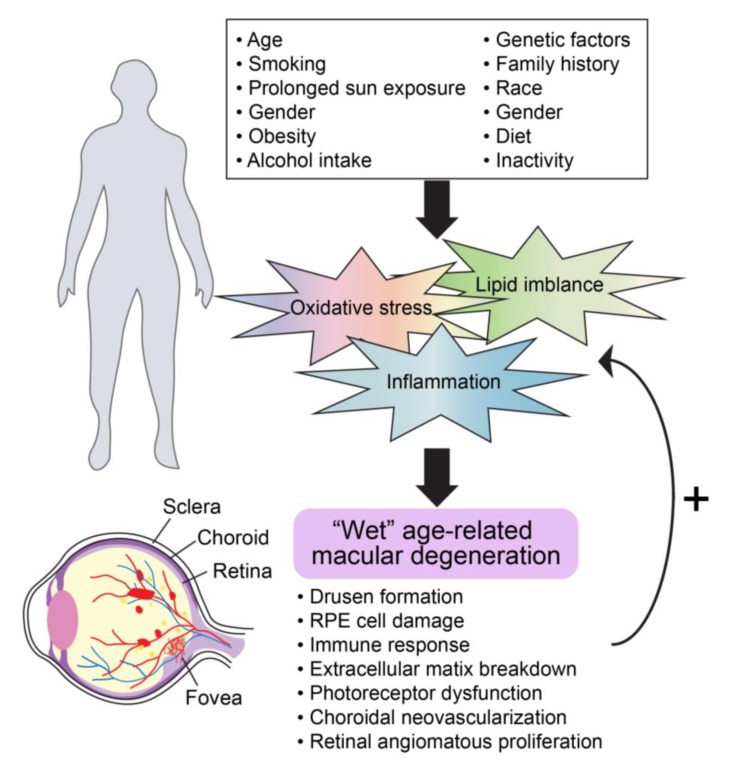
General molecular mechanisms involved in advanced AMD progression.

AMD is a progressive macular degenerative disease with complex and heterogeneous features. The progression of AMD is highly variable among individuals. We and others have previously reviewed some of the signaling pathways implicated in the pathogenesis of AMD along with potential risk factors for developing the disease [12,13,14,15,16]. The biological pathways identified to date include but are not limited to inflammation, oxidative stress, lipid dysregulation, and vascular compromise. These in combination with genetic risk, environmental factors, and overall health conditions further underscore the complexity of AMD (Figure 2). Likewise, functional vision in the posterior segment relies on multiple cell types, including RPE cells, photoreceptors, choroidal endothelial cells, macrophages, and microglia. Thus, it is credible that the dysfunction of some or all of these cells and/or an imbalance in cellular communication may promote AMD advancement. With this in mind, the priority for developing new therapies relies on further realizing the underlying mechanisms of the disease and identifying targetable signaling pathways.

AMD patients with CNV, which tend to develop severe vision loss, typically exhibit increased angiogenesis and neovascularization in the choroid with hemorrhage and fluid accumulation in the subretinal space, ultimately involving the retinal vasculature in the case of retinal angiomatous proliferation. These phenotypic changes may be the result of imbalanced vascular inflammation, impaired epithelial and/or endothelial cell migration and adhesion, abnormal cell proliferation, and/or dysregulated mitochondrial function [17,18]. Newly formed blood vessels recruit inflammatory cells which can produce inflammatory cytokines, chemokines, and growth factors responsible for promoting angiogenesis [19]. Although acute inflammation can serve as the immediate response against pathogenic infection, a chronic inflammatory condition tends to favor angiogenesis, leading to pathological complications of diseases, such as wet AMD. Whether or not the increased number of macrophages attracted to the retina and neovascular lesions indicates exacerbation of the disease or a repair process from the damage continues to be an area of intense investigation and likely will dependent upon the timing of the macrophage recruitment concomitant with the influence of adjacent resident cells within the microenvironment, such as RPE cells.

RPE cells play a central role in regulating CNV progression through their production/secretion of a number of angiogenic factors in response to environmental stimuli, including complement factors and growth factors such as vascular endothelial growth factor (VEGF). To date, targeting VEGF, a potent pro-angiogenic mediator, is still the standard treatment for neovascular AMD [20,21,22], and the efficacies of different anti-VEGF strategies have been extensively investigated. In general, VEGF-A stimulates the migration of endothelial cells and promotes vascular proliferation, required for angiogenesis under both physiological and pathological conditions. However, despite some success in vision improvement [23], an increasing number of body of studies have revealed unexpected complications of long term anti-VEGF-A therapy in treating ocular diseases. Human studies have shown altered retinal vascular immune cell homeostasis and RPE toxicity in a subset of patients [24,25], while mouse models in which VEGF is absent present with choriocapillary atrophy [26]. 

### 3.3. Nuclear Receptors and AMD Pathobiology

Nuclear receptors (NRs) have been shown to play a physiological role in RPE cells, which are susceptible to oxidative stress and inflammatory stimuli and of relevance; these are classified as ‘AMD-vulnerable cells’. We previously established an NR atlas of human RPE cells [27], highlighting potential candidate receptors relevant to AMD pathogenesis. Increasing evidence is emerging to appreciate the functional participation of NRs in modulating ocular diseases through multiple mechanisms [16,28,29,30]. To date, the interest in finding links between NRs and wet AMD development and progression are still relatively new but growing. In the following sub-sections, we focus on recent studies illustrating the significance of specific nuclear receptors in wet AMD and, in particular, their contribution to regulation of angiogenesis using in vitro cell cultures and transgenic and/or experimentally induced laser CNV animal models.

#### 3.3.1. Peroxisome Proliferator-Activated Receptors (PPARs)

PPARs are ligand-binding nuclear receptors that heterodimerize with their obligate binding partner, retinoid-x-receptors (RXRs), to regulate target gene expression levels through interactions with DNA response elements within specific promoter regions [31,32]. This family of nuclear receptors were first discovered in the early 1990s by researchers who noticed peroxisomal proliferation via treatment with rodent hepatocarcinogens [33]. The physiological importance of PPAR function is diffuse, and PPARs have demonstrated importance in a variety of diseases not limited to inflammatory, diabetic, cardiovascular, cancer, neurodegenerative, and ocular diseases [34]. They also control a variety of biological processes, including adipogenesis, cell proliferation and differentiation, lipid and glucose metabolism, inflammation, angiogenesis, and immune function [35]. Of relevance to ocular biology, PPAR isoforms (PPARα, PPARβ/δ, and PPARγ) are expressed in the choriocapillaris, choroidal endothelial cells, retinal endothelial cells, and RPE cells [36]. A series of recent works have linked a high dietary intake of omega-3 long-chain polyunsaturated fatty acids (ω-3 LCPUFAs), which are endogenous agonists of PPARs, with reduced ocular angiogenesis [37,38,39,40,41,42]. These studies demonstrate a protective role of activating PPARs against retinal diseases, including neovascular AMD.

In addition to its well-known function in lipid metabolism, PPARα also has a critical role in inflammation. Similar to most NRs, whether PPARα exhibits a pro- or anti-inflammatory effect is highly tissue- and cell-specific [35]. A recent study explored the effect of activating PPARα on ocular neovascularization using laser-induced CNV rat and transgenic mouse models [43]. The systemic administration of fenofibric acid (Feno-FA), a potent PPARα agonist, attenuated laser-induced CNV lesions and decreased inflammatory cytokine production. It is also worth noting that PPARα^−/−^ mice exhibit severe CNV features, and Feno-FA treatment has no effects on rescuing this phenotype [43]. 

In the *Ccl2*^−/−^/*Cx3cr1*^−/−^ mice, a potential neovascular model, PPARγ expression is significantly increased [36], suggesting a conceivable role for PPARγ in AMD. The inhibitory effect of activating PPARγ by troglitazone or rosiglitazone on VEGF-induced proliferation and migration, VEGF-induced angiogenesis in vitro, and CNV lesions in vivo, using cultured cells (e.g., human RPE cells and bovine choroidal endothelial cells) and in rats, respectively, suggests that targeting PPARγ may be beneficial for treating neovascular AMD patients [44]. Mechanistically, the PPARγ-dependent modulation of CNV progression may be attributable to PPARγ’s essential role in regulating the expression of genes associated with inflammatory and oxidative stress pathways [35,45].

Compared to PPARα and PPARγ, the biological roles of PPARβ/δ are considerably less defined. One potential mechanism of PPARβ/δ is that its activation may inhibit genetically or chemically induced inflammation in part by the reduced expression of cytokines through the trans-repression of NF-κB-dependent signaling [35]. On the other hand, the activation of PPARβ/δ may induce migration and angiogenesis in human endothelial cells, which is associated with the up-regulation of VEGF and matrix metalloproteinase 9 (MMP-9) [46]. With regard to AMD biology, *PPARβ/δ* is expressed in both RPE and choroidal endothelial cells [28], and aged *PPARβ/δ*^−/−^ mice have been shown to develop several features of early dry AMD, including thin continuous sub-RPE deposits, Bruch’s membrane thickening, RPE pigmentary changes, and disorganized basal infoldings in RPE cells, suggesting an essential role of PPARβ/δ in RPE cell health [28]. Importantly, knocking down *PPARβ/δ* expression or antagonizing PPARβ/δ activity has been shown to inhibit angiogenesis in vitro and attenuate the severity of laser-induced CNV lesions in vivo [28]. Thus, the functions of PPARβ/δ in pathogenic angiogenesis are most likely complicated, reflecting the need to consider cell-specific and selective modulation of PPARβ/δ in dry (receptor agonism) versus wet AMD (receptor antagonism). 

#### 3.3.2. Liver X Receptors (LXRs)

Closely related to the PPAR subfamily, the LXRs act as cholesterol sensors, regulating glucose and cholesterol homeostasis [28], inflammation, and central nervous system development in response to endogenous and/or exogenous lipid ligands [47]. Transactivation and transrepression are the two genomic mechanisms that drive downstream LXR gene transcription. Transactivation involves the heterodimerization of LXR with RXR followed by binding to LXR response elements, the shedding of corepressors, and the recruitment of coactivators [48]. Transrepression requires the monomeric sumoylation of lysines in the ligand-binding domain of LXR to tether the monomer to a multimolecular corepressor complex [49], the molecular understanding of which is mildly understood. Both LXR isoforms, alpha and beta (*LXRα* and *LXRβ*), are present in the retina, and their expression in human RPE-choroidal fractions decreases with age, which is the main risk factor of AMD [50,51]. *LXRα*^−/−^ mice exhibit a progressive accumulation of neutral lipid rich extracellular deposits underneath the RPE, representing a typical early dry AMD phenotype [50]. Furthermore, aged *LXRα*^−/−^ mice present with increased number of immune cells in the outer retina and elevated production of inflammatory cytokines within the RPE/choroid, which together reflect a pro-inflammatory response in the absence of *LXRα* [50]. In vitro, the ligand activation of LXR markedly suppresses the expression of inflammatory marker genes and attenuates intracellular lipid accumulation. The activation of LXRβ by a synthetic LXR ligand has been shown to protect the inner retinal damage against chemically-induced retinal degeneration. The protective role of LXRβ in ocular diseases is likely associated with the inhibited NF-κB signaling pathway and decreased amyloid-β formation as evidence in *LXRβ*^−/−^ mice [51,52]. Similarly, the activation of LXRα, in vivo, in the apoB-100 expressing mouse suppresses retinal inflammation and neutral lipid deposition in Bruch’s membrane [50]. Finally, there is evidence for the role of LXRs in regulating ocular angiogenesis and CNV pathogenesis. This comes from not only a genome-wide microarray analysis study indicating that the early suppression of VEGF ligand-receptor signaling and inflammatory pathways associated with corneal angiogenesis is coupled with the activation of LXR/RXR, PPARα/RXRα, and STAT3 pathways [53], but also in vivo studies in which treatment with an LXR agonist reduced the size and severity of laser induced CNV lesions in aged mice [54]. Given the fact that AMD patients often develop the dry form prior to neovascular AMD, dry AMD is considered a risk factor for developing wet AMD, and it is supposed that dry AMD treatments would also provide some protection against wet AMD [11].

#### 3.3.3. Estrogen Receptors (ERs)

The sex steroid hormone estrogen and its receptor (estrogen receptor, ER) regulate diverse signaling pathways involved in cell differentiation, cell migration, survival, cell death, and synaptic responses in neurons. There is evidence of estrogen production in the eye, as both *ERα* and *ERβ* have been detected in the human retina [55], suggesting an ocular physiological role of ER signaling. Gender-focused studies have revealed significantly higher CNV scores in experimentally induced laser neovascular formation in females versus male rats in conjunction with elevated expressions of *ERβ* and the VEGF receptor 2 [56]. These studies are consistent with gender differences in the incidence of neovascular AMD [57,58]. 

Changes in ERβ and VEGFR2 levels have also been observed in 17β-estradiol (E2)-treated ovariectomized females. Similarly, estrogen exacerbating CNV formation also occurs in the laser-induced CNV mouse model in association with elevated TNFα expression and the activation of macrophages [59]. More recently, single nucleotide polymorphisms in the *ERα* and matrix metalloproteinase 2 (*MMP2*) genes have been shown to be associated with neovascular AMD and in particular in women [60,61]. Adverse effects of exogenous estrogen on wet AMD progression raises the concern of estrogen and its function in vision [55,62]. However, studies also suggest that hormone replacement therapy (HRT) and/or oral contraceptives may exhibit protective effects in women against neovascular AMD [62,63,64]. More studies are needed to clarify the relationship between estrogen and AMD in the context of aging. Case in point, a recent study reported CNV in young adult females after hormonal treatment for ovarian stimulation during fertility therapy [65], while hormone treatment and the use of oral contraceptives at postmenopausal age are associated with a lower risk of neovascular AMD [64].

#### 3.3.4. Aryl Hydrocarbon Receptor (AhR)

Although not a traditional nuclear receptor, the aryl hydrocarbon receptor (AhR) also translocates to the nucleus upon ligand binding, interacts with the AhR nuclear translocator (ARNT) to form a heterodimer, and acts as transcriptional regulator to regulate xenobiotic metabolism, development, and carcinogenesis [66]. Previous studies have shown that the dysregulation of AhR fails to induce VEGF-dependent tube formation in human endothelial cells, and *AhR*^−/−^ mice exhibit impaired angiogenesis [67], which is indicative of a critical role of AhR in angiogenesis.

More recently, the differential regulation of AhR has been found to be associated with different AMD pathogeneses based on human RPE-choroid fractions subjected to high-throughput RNA sequencing [68]. Identified signaling pathways include inflammation, angiogenesis, and extracellular matrix regulation, supporting the functional significance of the AhR-mediated signaling pathway in AMD. We previously found that aged *AhR*^−/−^ mice spontaneously develop a dry AMD-like pathology, featuring thick sub-RPE deposit formation, disrupted RPE cell tight junctions, the accumulation of RPE cell lipofuscin, Bruch’s membrane thickening, and RPE and choroidal atrophy [69]. Interestingly, in the absence of *AhR*, aged mice following laser-induced CNV develop lesions larger in area and volume compared to age-matched wild-type mice [68]. These lesions are also associated with typical characteristics observed in human wet AMD, including an increased number of ionized calcium-binding adaptor molecule 1-positive (Iba1)-positive microglial cells and enhanced collagen type IV deposition, which is consistent with in vitro findings that knocking-down *AhR* increases the production of inflammatory cytokines and growth factors in RPE and choroidal endothelial cells. The activation of AhR by either leflunomide or flutamide significantly inhibits CNV formation in vitro and in vivo [70], demonstrating the therapeutic potential of targeting the AhR pathway in neovascular AMD. Thus, the differential regulation of AhR as either pro- or anti-angiogenic is cell- and tissue-dependent for nuclear receptors.

#### 3.3.5. Glucocorticoid Receptors (GR)

Glucocorticoids are essential steroid hormones that bind to glucocorticoid receptors (GRs) to regulate metabolic homeostasis. The anti-inflammatory and immunosuppressive ability of glucocorticoids underlie their critical roles in a large number of human medical conditions, including ocular diseases [71,72,73,74]. Upon ligand binding, GRs undergo conformational changes, translocate into the nucleus, and act as transcription factors by directly regulating target gene expression or by indirectly interfering with other transcription factor-mediated signaling pathways [75]. A recent study has revealed an interaction between GR- and AhR-mediated signals in ARPE19 cells [76], pointing to a novel molecular mechanism in RPE biology and potentially AMD. Injections or implants of dexamethasone or triamcinolone acetanoid (TA), synthetic glucocorticoids, are commonly used in suppressing neovascularization in both laser-induced CNV animal models and human studies [71,77,78,79]. Recent studies also demonstrate the power of combination therapy using verteporfin (photodynamic therapy), glucocorticoids, and anti-VEGF agents in choroidal neovascularization [80,81,82]. The use of triple therapy not only improves the visual acuity of CNV patients, but it also reduces the frequency of repeating treatments. Anecortave acetate, a glucocorticoid analogue, has been shown to reduce choroidal neovascularization without affecting normal retinal angiogenesis [83,84,85]. Interestingly, anecortave acetate does not trigger GR-mediated signaling [86], indicating a unique mechanism of action of glucocorticoids. 

### 3.4. Case Studies and Clinical Trials Examining the Relationship between Nuclear Receptors and AMD

There are a limited number of case studies and clinical trials that have either been completed or are in progress, examining associations between various nuclear receptors and AMD (Table 1). It is notable that several nuclear receptors are currently FDA approved, which may facilitate faster testing in man should preclinical studies provide support for targeting said pathway in AMD.

## 4. Nuclear Receptor Signaling in the Pathogenesis of Diabetic Retinopathy 

### 4.1. Overview of Diabetic Retinopathy

**Figure 3:**
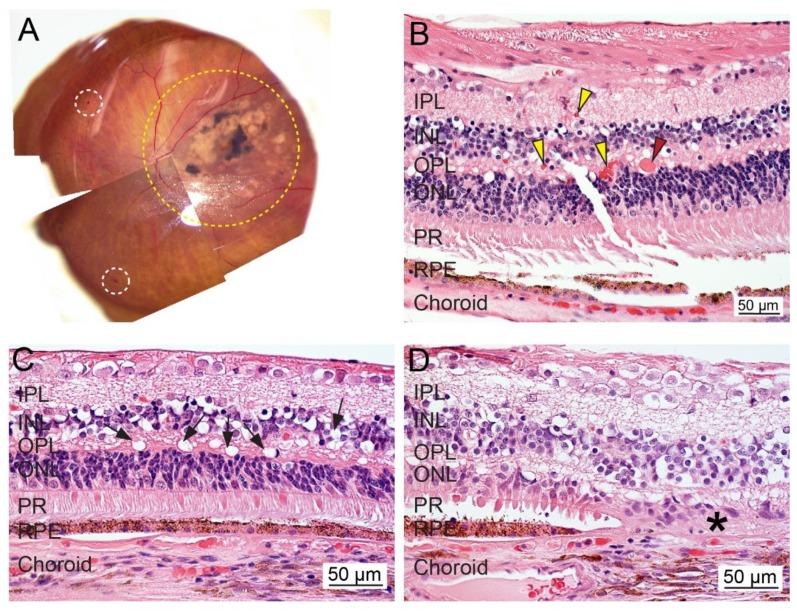
Ex vivo whole tissue and cross-sectional histopathology of human diabetic retinopathy (DR). (**A**) The representative fundus image of an eye from a patient diagnosed with DR, glaucoma, and AMD (OS; 70-year-old female; death to recovery: 4 h 35 min; cause of death: Parkinson’s). The area with severe lesions is delineated with a yellow dotted line. The white dotted circle indicates hemorrhages. (**B**–**D**) Representative photomicrographs of paraformaldehyde-fixed paraffin-embedded cross-sections of the retina stained with hematoxylin and eosin. Diabetic macular edema fluid was found in the retina (red arrowhead). Some areas showed intact RPE cells, while the retina contains increased infiltrated blood cells (yellow arrowheads) and exhibits vacuolization and atrophy (black arrows). Loss of photoreceptors and RPE cells is noted (asterisks). IPL, inner plexiform layer; INL, inner nuclear layer; OPL, outer plexiform layer; ONL, outer nuclear layer; PR, photoreceptor; RPE, retinal pigment epithelium. Bar = 50 µm.

**Figure 4:**
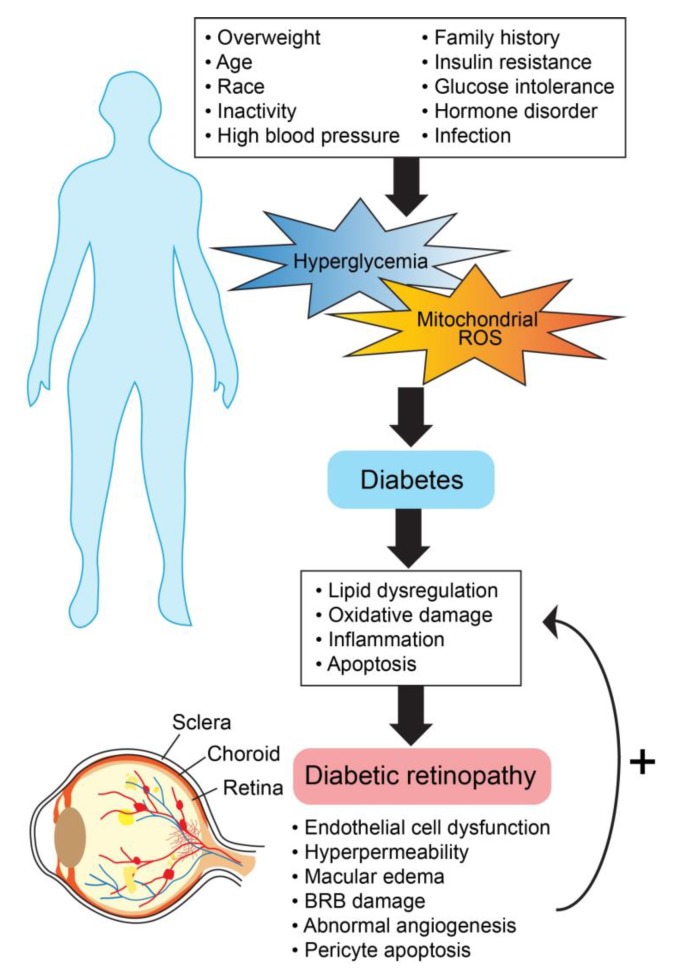
General molecular mechanisms involved in diabetic retinopathy progression.

Diabetic retinopathy (DR) impacts the lives of hundreds of millions across the globe [92]. Changes in the retinal vasculature, marked by physiologic and pathologic abnormalities (Figure 3), are vast, and yet abnormal vessel growth and macular edema remain the leading concerns related to vision loss [93]. Primary risk factors for DR include the duration of diabetes, hemoglobin A_1c_, hypertension, hyperlipidemia, and hyperglycemia [94,95,96]. Clinical findings for diabetic retinopathy present along a spectrum of severity, beginning with microaneurysm(s) (mild non-proliferative diabetic retinopathy; NPDR) and advancing to visible neovascularization and angiogenesis (proliferative diabetic retinopathy; PDR) [97]. The disease progression of DR at the molecular level is not well understood; still, several pathways have been shown to be highly involved in the process (Figure 4). These pathways are exacerbated by conditions of hyperglycemia and elevated mitochondrial reactive oxygen species (mROS), resulting in neurovascular damage dictated by oxidative stress, apoptosis, lipid dysregulation, and inflammation [98,99]. Our understanding of the molecular mechanisms is ever expanding. An area of promise is the involvement of nuclear receptors, namely PPARs, LXRs, vitamin D receptor (VDR), retinoic acid receptor-related orphan receptors (RORs), Rev-ErbAs, glucocorticoid receptor (GR), and mineralocorticoid receptor (MR).

### 4.2. Nuclear Receptors and Diabetic Retinopathy

#### 4.2.1. Peroxisome Proliferator-Activated Receptors (PPARs) 

PPARs are ubiquitously expressed in endothelial tissue types, making them a probable therapeutic target for angiogenic diseases [100]. As such, they have been under investigation in ocular diseases complicated by angiogenesis, including as mentioned earlier AMD, and as will be reviewed below, DR. Of the three sub-types, PPARα is most implicated in the underlying mechanisms of diabetic retinopathy. Hu et al. established the presence of PPARα in human and rat retina by immunofluorescent staining and found reduced expression levels in diabetic retinopathy human sections [101]. PPARα mRNA and protein expression levels were markedly reduced in diabetic animal models, including the STZ-induced diabetic rats, Akita mice, and db/db mice. These results were confirmed in several retinal cell lines (hTERT RPE cells, rMC-1 rat muller cells, and primary human retinal capillary pericytes) by treatment for 72 h with high glucose. In all models, PPARβ/δ and PPARγ expression levels were unchanged. Furthermore, it was found that PPAR-α knockout mice retained greater retinal vascular leakage, leukostasis, pericyte loss, capillary loss, capillary degeneration, and the over-expression of inflammatory markers. Of therapeutic relevance, fenofibrate, a potent PPARα agonist originally considered to have roles in lipid regulation [102], anti-inflammation [103,104], and anti-apoptosis [105], has shown promise in clinical trials as a possible oral treatment option for diabetic retinopathy by preventing microvascular complications [106]. In vivo, fenofibrate is rapidly converted to fenofibric acid by plasma and tissue esterases and consecutively binds to PPARα to promote the formation of PPARα-RXR heterodimers and downstream target gene expression [107]. In terms of angiogenic capacity, PPARα activation is known to inhibit SP1 activity, matrix metalloproteinases, VEGF, bFGF, LRP6 phosphorylation, WNT signaling, endothelial cell proliferation, and capillary tube formation while increasing TSP-1 and endostatin activity [107,108]. Taken together, PPAR-α is directly implicated in the progression of diabetic retinopathy. 

PPARβ/δ and PPARγ have also been identified as potential therapeutic targets for pathogenesis. The role of PPARβ/δ in ocular angiogenesis is mainly pro-angiogenic, yet contradictory evidence is reported in various non-ocular endothelial tissue types [108]. In vitro treatment of primary human retinal microvascular endothelial cells with GW0742, a PPARβ/δ agonist, and GSK0660, a PPARβ/δ antagonist, revealed dose–response increases and decreases in tube formation, respectively [109]. Results were complemented in vivo. Rat model intravitreal injections of GW0742 exacerbated preretinal neovascularization and GSK0660 prevented preretinal neovascularization, suggesting the potential role that PPARβ/δ could have in treating ocular angiogenesis. PPARγ is a well-characterized target for the insulin-sensitizing drugs called thiazolidinediones [110]. Key roles of PPARγ include glucose metabolism, inflammation, and angiogenesis [111]. Early studies have linked certain risk mutations in PPARγ with DR [112,113], and the presence of PPARγ in the vitreous and aqueous humor suggests its involvement in DR [114]. Conversely, PPARγ expression is suppressed in diabetes-induced streptozotocin wild-type mice and high glucose-treated human umbilical vein endothelial cells (HUVECs) [115].

#### 4.2.2. Liver-X-Receptors (LXR)

Lipid dysregulation is hallmark of DR, as such, LXR have also been studied extensively in the pathogenesis of the disease [116,117]. In a seminal study by Hazra et al., oxygen-induced retinopathy (OIR) mice were treated with GW3965, an LXR agonist. Compared to untreated mice, GW3965-treated OIR mice marked a 30% reduction in preretinal blood vessels, which is an end-point measurement of angiogenesis [118]. The mechanisms of action of LXR activation in DR have further been investigated, and it has been shown to restore reverse cholesterol metabolism, prevent inflammation, reduce pro-inflammatory macrophage activity, and prevent the formation of diabetes-induced acellular capillaries [116].

#### 4.2.3. Vitamin D Receptor

The vitamin D receptor (VDR) is another ligand-binding nuclear receptor with therapeutic relevance to the progression of angiogenesis in diabetic retinopathy. VDR is activated by 1,25-dihydroxyvitamin D_3_, a direct metabolite of vitamin D, and constitutively dimerizes with RXR to regulate target gene expression by binding vitamin D response elements [119]. The connection between VDR and DR is not well understood at the molecular level. However, Merrigan and Kennedy [120] utilized an unbiased phenotype screening protocol to identify small molecule regulators of ocular angiogenesis. Calcitriol, an agonist of VDR, was identified and tested alongside other known VDR agonists (calcipotriol, seocalcitrol, and maxacalcitrol) in zebrafish. VDR activation suggested an anti-angiogenic response, which may help to explain the association between VDR and DR.

#### 4.2.4. Retinoic Acid Receptor (RORs) and Rev-Erbs

Retinoic acid receptor-related orphan receptors (RORs) and Rev-ErbAs are two nuclear receptors that share DNA-binding homology at ROR response elements, and in many cases antagonize each other [121,122,123]. The main structural difference between these two nuclear receptors is the presence of activation function 2 (AF-2) regions in RORs and absence in Rev-ErbAs [124]. The lack of AF-2 in Rev-ErbA allows for the recruitment of repressors such as nuclear repressor corepressor (NCOR) [122]. The natural ligand(s) for RORs are controversial, but there is some consensus that oxysterols are high affinity substrates, similar to LXRs [125,126]. On the other hand, the natural ligand for Rev-ErbAs is heme [123]. 

In an ischemia-induced mouse model, homozygous staggerer mutant mice (*ROR^sg/sg^*) were compared to wild type C57BL/6 mice and demonstrated a 2-fold increase in angiographic score, 3-fold increase in capillary density, increases in eNOS expression levels, and decreases in IL-12 levels [127]. Interestingly, other ocular studies indicate RORα to have a proangiogenic role by suppressing target genes suppressors of cytokine signaling 3 (SOCS3) and semaphorin 3E (Sema-3E) in OIR and angiogenic mouse models [128,129]. SOCS3 and Sema-3E are established antiangiogenic factors. SOCS3 acts as an endogenous inhibitor of pathologic angiogenesis by regulating inflammation and growth factor signaling [130], and Sema-3E-PlexinD1 signaling helps orchestrate new blood vessels formation specifically toward ischemic regions while retaining antiangiogenic capabilities [131,132,133].

Importantly, several studies have identified RORs and Rev-ErbAs as regulators of circadian rhythm, lipid homeostasis, and angiogenesis [122,134]. Others suggest that an interconnected relationship may exist between these functions and systemic and ocular-related vascular diseases [128,135,136,137,138,139]. In a rat model of diabetic mellitus type 2, Busik et al. discovered DR to be a downstream consequence to bone marrow neuropathy succeeded by decreases in endothelial progenitor cell (EPC) number, migratory potential, and reparative capacity. Changes in EPC function were dictated by decreases in circadian patterns and expression levels of circadian-related genes, including RORα [140].

#### 4.2.5. Mineralocorticoid Receptors (MR) and Glucocorticoid Receptors (GR) 

MR and GRs are nuclear receptors that share binding potential with aldosterone, cortisol, and corticosterone [141]. Targeting these NRs with steroids has been used in the treatment of systemic vascular diseases and has shed light onto its use in diabetic retinopathy [142,143]. In a rat model of OIR and OIR-stimulated bovine retinal endothelial cells, treatment with spironolactone, an antagonist of MR, was found to be antiangiogenic. Furthermore, protein and mRNA levels of genes involved in retinal inflammation, leukostasis, and monocyte chemoattractant-1 were reduced [144]. 

While our understanding of GR is not well-established at the molecular level, glucocorticoids are a common treatment method for diabetic macular edema. Glucocorticoids activate GR and protect the blood–retina barrier by increasing endothelial cell tight junctions [145]. Evidence also suggests that glucocorticoids may act on GR to inhibit VEGF expression and angiogenic capacity [146].

### 4.3. Human Studies Examining the Potential Role of Nuclear Receptors in Diabetic Retinopathy.

Similar to AMD, there are a number of case studies and clinical trials that have either been completed (Table 2) or are ongoing intending on determining the therapeutic potential of nuclear receptors in diabetic retinopathy.

## 5. The Future of Nuclear Receptor Targeted Therapies for Ocular Neovascular Diseases

Abnormal angiogenesis is a common denominator among many systemic and neurodegenerative diseases. The plethora of studies on the role of nuclear receptors in vasculogenesis and angiogenesis in non-ocular tissues dictates the need to explore their potential role in the eye. Although studying NR biology in eye diseases is relatively new, herein, we presented a brief summary of the studies to date that have demonstrated the value of investigating the role of nuclear receptors in ocular angiogenesis and vascular disease. These studies are continuing and ever expanding to other ocular vascular diseases not covered in this review due to space limitations, including but not limited to the retinopathy of prematurity, in which RORs, PPARs and ERs have been shown to play a role and central serous chorioretinopathy in which MRs have been implicated. Overall, it is clear that the role of NRs in ocular diseases, similar to other tissues, is diverse and complicated; however, success in harnessing the power of NR targeting in the eye will continue to improve with growing access to relevant in vitro and in vivo models as well as new nuclear receptor pharmacological agents including selective NR modulators.

## Figures and Tables

**Table 1:** Case studies or clinical trials examining the relationships between nuclear receptors and wet AMD. CNV: choroidal neovascularization, GR: glucocorticoid receptor, ERs: estrogen receptors, PPAR: peroxisome proliferator-activated receptors, VEGF: vascular endothelial growth factor.

Reference	Target	Type of Study	Cohort Size	Results and Interpretation
Hong et al., 2018. Review [87]	PPARs	unknown	unknown	PPARα agonist macuneos (Biophytis) is under clinical trial phase I for treating AMD.
The Eye Disease Case–control Study Group. 1992 [88]	ERs	Case-control study	*n* = 1036	Women in the U.S. exposed to exogenous estrogen exhibited lower risk of neovascular AMD.
Snow et al., 2002 [64]	ERs	Cross-sectional study on postmenopausal women with AMD	*n* = 394	Women under postmenopausal estrogen therapy experienced lower grade of AMD.
Tomany et al., 2004 [89]	ERs	Population-based cohort study (meta-analysis)	*n* = 9523	No significant associations between the use of hormone therapy and the incidence of late AMD was reported.
Boekhoorn et al., 2007. The Rotterdam Study [60]	ERs	Population-based cohort study	*n* = 4571	ERα polymorphisms (*ESR1 PvuII-XbaI*) are associated with an increased incidence of wet AMD.
Edwards et al., 2010 [63]	ERs	Case-control study	*n* = 799	Hormone replacement therapy or oral contraceptives have a protective role in women with neovascular AMD.
Spaide et al., 2005 [90]	GRs	Small cohort study	*n* = 26	CNV patients treated with combined photodynamic therapy with verteporfin and intravitreal triamcinolone acetonide (GR agonist) exhibited improved vision and reduced treatment frequency.
Augustin et al., 2007 [81]	GRs	Small cohort study	*n* = 104	One cycle of triple therapy with verteporfin (photodynamic therapy), dexamethasone (GR agonist), and bevacizumab (anti-VEGF) improved the visual acuity of CNV patients.
Ehmann et al., 2010 [82]	GRs	Small cohort study	*n* = 30	One cycle of triple therapy with verteporfin (photodynamic therapy), dexamethasone (GR agonist), and bevacizumab (anti-VEGF) improved visual acuity of CNV patients.
Gallemore et al., 2017. The RADICAL Study [91]	GRs	Randomized control study	*n* = 162	Combined therapy with verteporfin (photodynamic therapy), ranibizumab (anti-VEGF) and dexamethasone (GR agonist) significantly reduced retreatment visits than ranibizumab treatment alone in CNV patients.
Capuano et al., 2019 [79]	GRs	Small cohort study	*n* = 3	Intravitreal implants of dexamethasone (GR agonist) improved the vision of pregnant CNV patients.

**Table 2:** Summary of relevant NRs in human studies of DR.

Reference	Target	Type of Study	Study size	Results and Interpretation
ACCORD Study Group et al., 2014 [106]	PPARα	Randomized, controlled clinical trial	*n*= 1593 type II diabetes mellitus patients (806 fenofibrate treatment; 787 placebo)	Patients treated with fenofibrate, a potent PPARα agonist, were less likely to develop diabetic retinopathy (adjusted OR = 0.60; 95% CI 0.42–0.87; *p* = 0.006).
Costa V et al., 2009 [112]	PPARγ	Case control	*n* = 670 (211 type II diabetes; 205 obese; 254 control individuals)	Pro12Ala polymorphism of the *PPARγ* gene may be associated with decreased risk of DR.
Malecki MT et al., 2008 [113]	PPARγ	Case control	*n* = 159 (38 type II diabetes without DR; 121 with DR)	Polymorphism A-2819 in the *PPARγ* gene is associated with DR.
Taverna et al., 2002 [147]	VDR	Cross-sectional	*n* = 200 c-peptide negative type I diabetics	Homozygous wild-type (TT) individuals had lower odds of “severe” diabetic retinopathy (OR = 0.50; 95% CI, 0.26–0.94; *p* = 0.02).
Taverna et al., 2005 [148]	VDR	Cross-sectional	*n* = 254 c-peptide negative type I diabetics	Those with severe DR were less likely to have the FF genotype than those individuals with none or mild DR (OR = 0.54; 95% CI, 0.32–0.90).
Cyganek et al., 2006 [149]	VDR	Cross-sectional	*n* = 267 type II diabetics	FOKI, TAQI, BSMI, and APA1 polymorphisms of VDR were not associated with DR.
Bućan et al., 2009 [150]	VDR	Cross-sectional	*n* = 120 type I diabetics	FOKI, TAQI, and TRU91 polymorphisms were not significantly associated with DR. BSMI was weakly associated with DR (*n* = 7, *p* < 0.05).
Jia et al., 2015 [151]	VDR	Case control	Cases = 81 Controls = 113	TAQI T allele (OR = 2.78; 95% CI, 1.15–6.72) and BSMI b allele (OR = 3.20; 95% CI, 1.19–8.60) in VDR gene are associated with diabetic retinopathy.
